# Supplementary material for: Drug repurposing for aging research using model organisms
Source: Aging Cell. 2017 Jun 16;16(5):1006–15. doi: 10.1111/acel.12626 (PMC5595691; doi:10.1111/acel.12626)
Supplement: Supplementary file 7 — Data S1 Zip‐Archive of all report cards. [file ACEL-16-1006-s007.zip › RC_NAI.pdf]

NAI

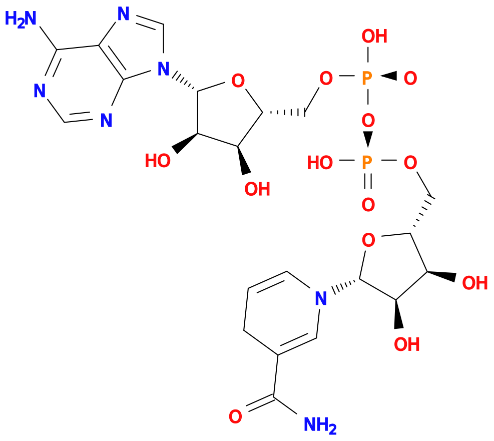

Database identifiers

DrugBank	DB00157
CHEBI	16908
eMolecules	36366750

Ranking

	Rank	Score
Drosophila	176/697	0.722
C. elegans	332/591	0.178

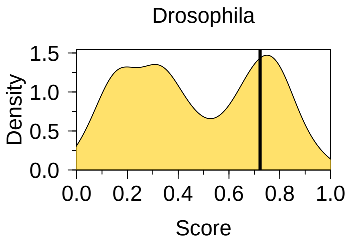

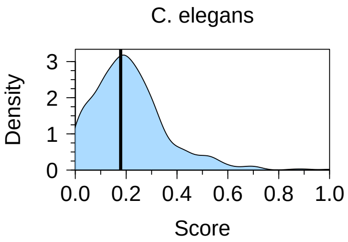

	Ageing implication	Domain conservation	Binding site conservation	Binding affinity	Bioavailability	Lipinski	Promiscuity	Purchasability	Drug approval	**Total**
Drosophila	1.0	0.955	0.922	0.893	(0.9)	-0.15	-0.009	0.1	0.075	0.722
C. elegans	1.0	0.957	0.94	0.893	0.203	-0.15	-0.009	0.1	0.075	0.178

Names

- **NADH**
- 1,4-dihydronicotinamide adenine dinucleotide
- DPNH
- nicotinamide adenine dinucleotide (reduced)
- Reduced nicotinamide adenine dinucleotide

Roles

fundamental metabolite

Status

Classification (according to DrugBank)	nutraceutical
Administration Route	Oral
Number of Rule of 5 violations	3
Binding affinity to original target in log units (RF-Score prediction)	7.12
Burns *C. elegans* bioavailability prediction	-8.69

Medical Information

Indicatation: Some evidence suggests that NADH might be useful in treating Parkinson's disease, chronic fatigue syndrome, Alzheimer's disease and cardiovascular disease.
Mechanism of action: NADH is synthesized by the body and thus is not an essential nutrient. It does require the essential nutrient nicotinamide for its synthesis, and its role in energy production is certainly an essential one. In addition to its role in the mitochondrial electron transport chain, NADH is produced in the cytosol. The mitochondrial membrane is impermeable to NADH, and this permeability barrier effectively separates the cytoplasmic from the mitochondrial NADH pools. However, cytoplasmic NADH can be used for biologic energy production. This occurs when the malate-aspartate shuttle introduces reducing equivalents from NADH in the cytosol to the electron transport chain of the mitochondria. This shuttle mainly occurs in the liver and heart.
Toxicity: No reports of overdose, however, high doses of NADH (10 mg a day or more) may cause jitteriness, anxiety, and insomnia.
Retail: Spectrum Pharmaceuticals
Nicotinamide adenine powder - 154.53 USD / g

(Information from DrugBank)

Compound Target Characteristics

Dihydrolipoyl dehydrogenase, mitochondrial

Best gene implication in ageing for this target family came from gene O17953 via mapping the annotation from WormBase WBGene00010794 annotated in WormBase 2014-03-11. Annotation GO 8340 (determination of adult lifespan) was Inferred from Mutant Phenotype

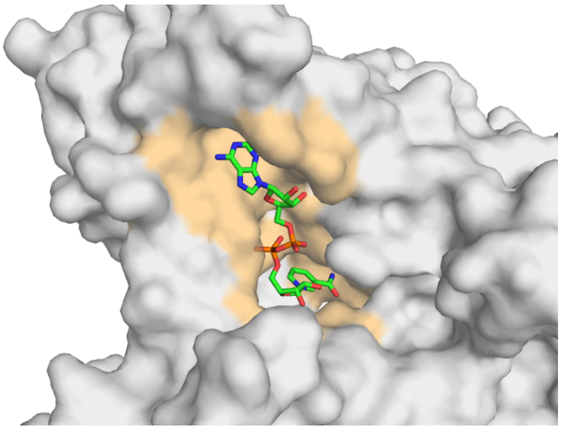

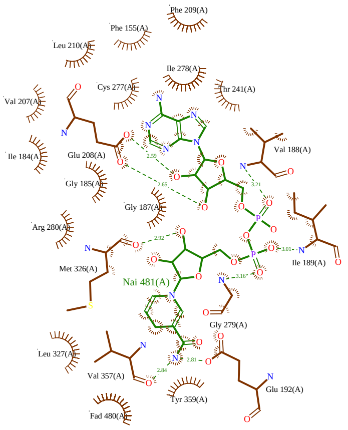

protein	amino acids contacts (binding site)
PDB:1zmd:chainA:P09622	K F I G G V I E V E F L T V C I G R M L V Y
tr:E9PEX6:E9PEX6_HUMAN	K F I G G V I E V E F L T V C I G R M L V Y
sp:P09622:DLDH_HUMAN	K F I G G V I E V E F L T V C I G R M L V Y
sp:Q6P6R2:DLDH_RAT	K F I G G V I E V E F L T V C I G R M L V Y
sp:O08749:DLDH_MOUSE	K F I G G V I E V E F L T V C I G R M L V Y
tr:Q9VVL7:Q9VVL7_DROME	K F I G G V I E I E F M T V S V G R M L V Y
sp:O17953:DLDH_CAEEL	K F I G G V I E V E F L T V S V G R M L V Y
sp:P09624:DLDH_YEAST	K F I G G I I E V E F Q T V A V G R M L V Y

	whole protein		domain-based		contact-based	
protein	ident	simil	ident	simil	ident	simil
PDB:1zmd:chainA:P09622	1.0	1.0	1.0	1.0	1.0	1.0
tr:E9PEX6:E9PEX6_HUMAN	0.95	0.95	0.99	0.99	1.0	1.0
sp:P09622:DLDH_HUMAN	1.0	1.0	1.0	1.0	1.0	1.0
sp:Q6P6R2:DLDH_RAT	0.95	0.99	0.95	0.99	1.0	1.0
sp:O08749:DLDH_MOUSE	0.95	0.99	0.95	0.99	1.0	1.0
tr:Q9VVL7:Q9VVL7_DROME	0.65	0.86	0.69	0.9	0.82	0.92
sp:O17953:DLDH_CAEEL	0.68	0.88	0.71	0.91	0.91	0.94
sp:P09624:DLDH_YEAST	0.53	0.82	0.56	0.85	0.82	0.85

CG7430 (UniProt:Q9VVL7) annotation

Cofactor: FAD Evidence=(RuleBase:RU003692); Note=Binds 1 FAD per subunit. (RuleBase:RU003692);
Miscellaneous: The active site is a redox-active disulfide bond. (RuleBase:RU003692).

(Information from UniProt)

dld-1 (WBGene00010794) associated phenotypes

lethal, sterile

(Information from WormBase)

dld-1 (UniProt:O17953) annotation

Cofactor: FADNote=Binds 1 FAD per subunit. ;
Subcellular location: Mitochondrion matrix
Miscellaneous: The active site is a redox-active disulfide bond.

(Information from UniProt)

5'-AMP-activated protein kinase subunit gamma

Best gene implication in ageing for this target family came from gene P12904 annotated in UniProt release 2014_02. Annotation GO subterm of 7568 (aging) was Inferred from Mutant Phenotype

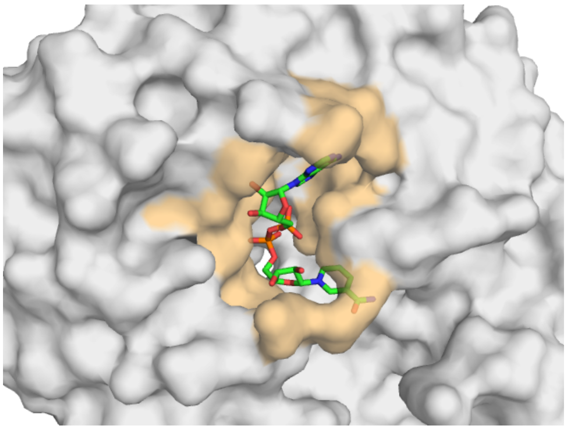

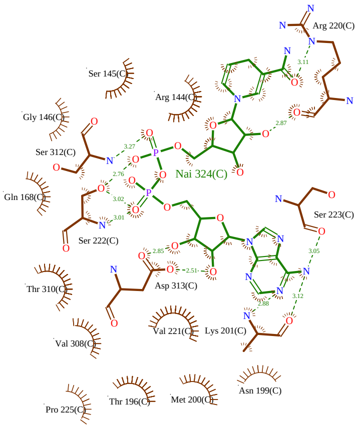

protein	amino acids contacts (binding site)																	
PDB:3te5:chainC:P12904	R	S	G	Q	T	N	M	K	R	V	S	S	P	V	T	L	S	D
tr:B4DT70:B4DT70_HUMAN	R	I	H	H	T	D	L	A	R	V	S	A	P	V	S	L	S	D
sp:Q9UGI9:AAKG3_HUMAN	R	I	H	H	T	D	L	A	R	V	S	A	P	V	S	L	S	D
tr:D4A7E4:D4A7E4_RAT	R	I	H	H	T	D	L	A	R	V	S	A	P	V	S	L	S	D
sp:Q8BGM7:AAKG3_MOUSE	R	I	H	H	T	D	L	A	R	V	S	A	P	V	S	L	S	D
tr:Q7KNS9:Q7KNS9_DROME	R	I	H	H	T	N	I	E	R	V	S	A	P	I	S	L	S	D
tr:Q8IGX0:Q8IGX0_DROME	R	I	H	H	T	N	I	E	R	V	S	A	P	I	S	L	S	D
tr:Q0KI34:Q0KI34_DROME	R	I	H	H	T	N	I	E	R	V	S	A	P	I	S	L	S	D
tr:A1A703:A1A703_DROME	R	I	H	H	T	N	I	E	R	V	S	A	P	I	S	L	S	D
tr:A1A6Y3:A1A6Y3_DROME	R	I	H	H	T	N	I	E	R	V	S	A	P	I	S	L	S	D
tr:O96613:O96613_DROME	R	I	H	H	T	N	I	E	R	V	S	A	P	I	S	L	S	D
tr:Q8I0K6:Q8I0K6_DROME	R	I	H	H	T	N	I	E	R	V	S	A	P	I	S	L	S	D
tr:Q8I0A1:Q8I0A1_DROME	R	I	H	H	T	N	I	E	R	V	S	A	P	I	S	L	S	D
tr:Q8SXT8:Q8SXT8_DROME	R	I	H	H	T	N	I	E	R	V	S	A	P	I	S	L	S	D
tr:Q8I0B2:Q8I0B2_DROME	R	I	H	H	T	N	I	E	R	V	S	A	P	I	S	L	S	D
tr:Q7JQX9:Q7JQX9_DROME	R	I	H	H	T	N	I	E	R	V	S	A	P	I	S	L	S	D
tr:Q9VDD2:Q9VDD2_DROME	R	I	H	H	T	N	I	E	R	V	S	A	P	I	S	L	S	D
tr:Q9BHL6:Q9BHL6_CAEEL	K	V	H	H	A	D	I	L	R	V	S	A	P	V	S	L	S	D
sp:P12904:AAKG_YEAST	R	S	G	Q	T	N	M	K	R	V	S	S	P	V	T	L	S	D
tr:F4ZZV2:F4ZZV2_YEASX	R	S	G	Q	T	N	M	K	R	V	S	S	P	V	T	L	S	D

	whole protein		domain-based		contact-based	
protein	ident	simil	ident	simil	ident	simil
PDB:3te5:chainC:P12904	1.0	1.0	1.0	1.0	1.0	1.0
tr:B4DT70:B4DT70_HUMAN	0.3	0.69	0.31	0.71	0.56	0.71
sp:Q9UGI9:AAKG3_HUMAN	0.2	0.49	0.31	0.74	0.56	0.71
tr:D4A7E4:D4A7E4_RAT	0.2	0.49	0.31	0.75	0.56	0.71
sp:Q8BGM7:AAKG3_MOUSE	0.2	0.49	0.31	0.75	0.56	0.71
tr:Q7KNS9:Q7KNS9_DROME	0.21	0.47	0.33	0.73	0.56	0.73
tr:Q8IGX0:Q8IGX0_DROME	0.19	0.45	0.33	0.75	0.56	0.73
tr:Q0KI34:Q0KI34_DROME	0.2	0.45	0.33	0.75	0.56	0.73
tr:A1A703:A1A703_DROME	0.17	0.39	0.33	0.75	0.56	0.73
tr:A1A6Y3:A1A6Y3_DROME	0.17	0.39	0.33	0.75	0.56	0.73
tr:O96613:O96613_DROME	0.16	0.37	0.33	0.75	0.56	0.73
tr:Q8I0K6:Q8I0K6_DROME	0.15	0.34	0.33	0.75	0.56	0.73
tr:Q8I0A1:Q8I0A1_DROME	0.13	0.3	0.33	0.75	0.56	0.73
tr:Q8SXT8:Q8SXT8_DROME	0.12	0.27	0.33	0.75	0.56	0.73
tr:Q8I0B2:Q8I0B2_DROME	0.12	0.27	0.33	0.75	0.56	0.73
tr:Q7JQX9:Q7JQX9_DROME	0.11	0.26	0.33	0.75	0.56	0.73
tr:Q9VDD2:Q9VDD2_DROME	0.08	0.17	0.33	0.75	0.56	0.73
tr:Q9BHL6:Q9BHL6_CAEEL	0.17	0.41	0.32	0.74	0.44	0.68
sp:P12904:AAKG_YEAST	1.0	1.0	1.0	1.0	1.0	1.0
tr:F4ZZV2:F4ZZV2_YEASX	1.0	1.0	1.0	1.0	1.0	1.0

SNF4Agamma (FBgn0264357) associated phenotypes

feeding behavior defective, hyperactive, neuroanatomy defective, nutrition conditional, phototaxis defective, short lived, starvation stress response defective

(Information from FlyBase)

NAD-dependent malic enzyme, mitochondrial

Best gene implication in ageing for this target family came from gene Q9NIW0 annotated in UniProt release 2014_02. Annotation GO 8340 (determination of adult lifespan) was Inferred from Mutant Phenotype

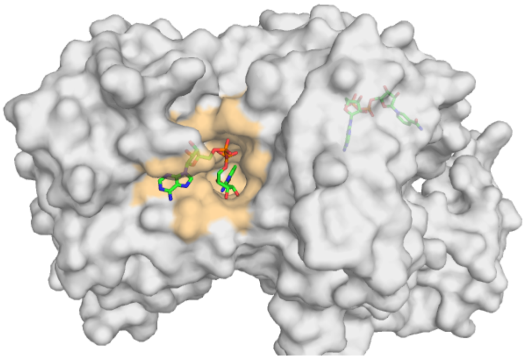

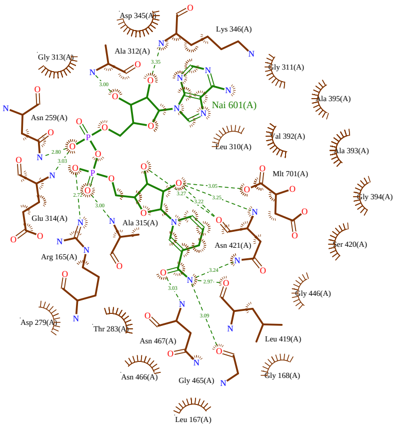

protein	amino acids contacts (binding site)
PDB:1pj2:chainA:P23368	H K R L G G I R R R Y G N D T L G A G E A D K V A G A L S N G G N N I L N R Y
sp:P23368:MAOM_HUMAN	H K R L G G I R R R Y G N D T L G A G E A D K V A G A L S N G G N N I L N R Y
tr:D3ZJH9:D3ZJH9_RAT	H K R L G G I Q K R Y G N D T L G A G E A D K V A G A L S N G G N N I L E R Y
sp:Q99KE1:MAOM_MOUSE	H K R L G G I Q K R Y G N D T L G A G E A D K V A G A L S N G G N N I L E R Y
tr:H7BWR6:H7BWR6_DROME	D R R L G G I K Q R F G N D T L G A G E A D S A A A Q L S N G G N N L C G V F
tr:Q9NIW0:Q9NIW0_DROME	D R R L G G I K Q R F G N D T L G A G E A D S A A A Q L S N G G N N L C G V F
tr:H7BWR4:H7BWR4_DROME	D R R L G G I K Q R F G N D T L G A G E A D S A A A Q L S N G G N N L C G V F
tr:H7BWU6:H7BWU6_DROME	D R R L G G I K Q R F G N D T L G A G E A D S A A A Q L S N G G N N L C G V F
tr:H7BWS3:H7BWS3_DROME	D R R L G G I K Q R F G N D T L G A G E A D S A A A Q L S N G G N N L C G V F
tr:H7BWS2:H7BWS2_DROME	D R R L G G I K Q R F G N D T L G A G E A D S A A A Q L S N G G N N L C G V F
tr:Q2QBM1:Q2QBM1_DROME	D R R L G G I K Q R F G N D T L G A G E A D S A A A Q L S N G G N N L C G V F
tr:H7BWS7:H7BWS7_DROME	D R R L G G I K Q R F G N D T L G A G E A D S A A A Q L S N G G N N L C G V F
tr:H7BWS6:H7BWS6_DROME	D R R L G G I K Q R F G N D T L G A G E A D S A A A Q L S N G G N N L C G V F
tr:H7BWU8:H7BWU8_DROME	D R R L G G I K Q R F G N D T L G A G E A D S A A A Q L S N G G N N L C G V F
tr:Q9U1J1:Q9U1J1_DROME	D R R L G G I K Q R F G N D T L G A G E A D S A A A Q L S N G G N N L C G V F
tr:Q9NIW1:Q9NIW1_DROME	D R R L G G I K Q R F G N D T L G A G E A D S A A A Q L S N G G N N L C G V F
tr:Q9VG32:Q9VG32_DROME	D R R L G G I K Q R F G N D T L G A G E A D S A A A Q L S N G G N N L C G V F
tr:Q9NIW2:Q9NIW2_DROME	D R R L G G I K Q R F G N D T L G A G E A D S A A A Q L S N G G N N L C G V F
tr:Q9VG31:Q9VG31_DROME	D R R L G G I K Q R F G N D T L G A G E A D S A A A Q L S N G G N N L C G V F
tr:Q9U296:Q9U296_CAEEL	N R R L G G I R W R F G N D T L G A G G A D I A S T V L S N G G N N V L R L F
sp:P36013:MAOM_YEAST	D D G L G G I H R V Y - V D T Y G A G S A D R C S T Q L S N G N N N V L R I W

protein	whole protein ident	simil	domain-based ident	simil	contact-based ident	simil
PDB:1pj2:chainA:P23368	1.0	1.0	1.0	1.0	1.0	1.0
sp:P23368:MAOM_HUMAN	1.0	1.0	1.0	1.0	1.0	1.0
tr:D3ZJH9:D3ZJH9_RAT	0.89	0.97	0.9	0.97	0.93	0.97
sp:Q99KE1:MAOM_MOUSE	0.88	0.96	0.9	0.97	0.93	0.97
tr:H7BWR6:H7BWR6_DROME	0.47	0.77	0.5	0.82	0.63	0.77
tr:Q9NIW0:Q9NIW0_DROME	0.46	0.76	0.5	0.82	0.63	0.77
tr:H7BWR4:H7BWR4_DROME	0.46	0.76	0.5	0.82	0.63	0.77
tr:H7BWU6:H7BWU6_DROME	0.45	0.76	0.5	0.82	0.63	0.77
tr:H7BWS3:H7BWS3_DROME	0.46	0.76	0.5	0.82	0.63	0.77
tr:H7BWS2:H7BWS2_DROME	0.46	0.76	0.5	0.82	0.63	0.77
tr:Q2QBM1:Q2QBM1_DROME	0.46	0.76	0.5	0.82	0.63	0.77
tr:H7BWS7:H7BWS7_DROME	0.45	0.76	0.5	0.82	0.63	0.77
tr:H7BWS6:H7BWS6_DROME	0.45	0.76	0.5	0.82	0.63	0.77
tr:H7BWU8:H7BWU8_DROME	0.46	0.76	0.5	0.82	0.63	0.77
tr:Q9U1J1:Q9U1J1_DROME	0.44	0.74	0.48	0.8	0.63	0.77
tr:Q9NIW1:Q9NIW1_DROME	0.36	0.61	0.5	0.82	0.63	0.77
tr:Q9VG32:Q9VG32_DROME	0.36	0.61	0.5	0.82	0.63	0.77
tr:Q9NIW2:Q9NIW2_DROME	0.36	0.61	0.5	0.82	0.63	0.77
tr:Q9VG31:Q9VG31_DROME	0.36	0.61	0.5	0.82	0.63	0.77
tr:Q9U296:Q9U296_CAEEL	0.47	0.78	0.51	0.84	0.63	0.76
sp:P36013:MAOM_YEAST	0.3	0.65	0.34	0.74	0.5	0.6

UniProt:H7BWR6 annotation
Cofactor: Mg(2+) Evidence=(PIRSR:PIRSR000106-3); Name=Mn(2+); Xref=ChEBI:CHEBI:29035; Evidence=(PIRSR:PIRSR000106-3); Note=Divalent metal cations. Prefers magnesium or manganese. (PIRSR:PIRSR000106-3);

(Information from UniProt)

Men (FBgn0002719) associated phenotypes
fast, intermediate, long lived, poor, sleep defective

(Information from FlyBase)

Men (UniProt:Q9NIW0) annotation
Cofactor: Mg(2+) Evidence=(PIRSR:PIRSR000106-3); Name=Mn(2+); Xref=ChEBI:CHEBI:29035; Evidence=(PIRSR:PIRSR000106-3); Note=Divalent metal cations. Prefers magnesium or manganese. (PIRSR:PIRSR000106-3);

(Information from UniProt)

UniProt:H7BWR4 annotation
Cofactor: Mg(2+) Evidence=(PIRSR:PIRSR000106-3); Name=Mn(2+); Xref=ChEBI:CHEBI:29035; Evidence=(PIRSR:PIRSR000106-3); Note=Divalent metal cations. Prefers magnesium or manganese. (PIRSR:PIRSR000106-3);

(Information from UniProt)

UniProt:H7BWU6 annotation
Cofactor: Mg(2+)Name=Mn(2+); Xref=ChEBI:CHEBI:29035; Note=Divalent metal cations. Prefers magnesium or manganese.;

(Information from UniProt)

UniProt:H7BWS3 annotation
Cofactor: Mg(2+) Evidence=(PIRSR:PIRSR000106-3); Name=Mn(2+); Xref=ChEBI:CHEBI:29035; Evidence=(PIRSR:PIRSR000106-3); Note=Divalent metal cations. Prefers magnesium or manganese. (PIRSR:PIRSR000106-3);

(Information from UniProt)

UniProt:H7BWS2 annotation
Cofactor: Mg(2+) Evidence=(PIRSR:PIRSR000106-3); Name=Mn(2+); Xref=ChEBI:CHEBI:29035; Evidence=(PIRSR:PIRSR000106-3); Note=Divalent metal cations. Prefers magnesium or manganese. (PIRSR:PIRSR000106-3);

(Information from UniProt)

Men (UniProt:Q2QBM1) annotation
Cofactor: Mg(2+)Name=Mn(2+); Xref=ChEBI:CHEBI:29035; Note=Divalent metal cations. Prefers magnesium or manganese.;

(Information from UniProt)

UniProt:H7BWS7 annotation
Cofactor: Mg(2+) Evidence=(PIRSR:PIRSR000106-3); Name=Mn(2+); Xref=ChEBI:CHEBI:29035; Evidence=(PIRSR:PIRSR000106-3); Note=Divalent metal cations. Prefers magnesium or manganese. (PIRSR:PIRSR000106-3);

(Information from UniProt)

UniProt:H7BWS6 annotation
Cofactor: Mg(2+) Evidence=(PIRSR:PIRSR000106-3); Name=Mn(2+); Xref=ChEBI:CHEBI:29035; Evidence=(PIRSR:PIRSR000106-3); Note=Divalent metal cations. Prefers magnesium or manganese. (PIRSR:PIRSR000106-3);

(Information from UniProt)

UniProt:H7BWU8 annotation
Cofactor: Mg(2+) Evidence=(PIRSR:PIRSR000106-3); Name=Mn(2+); Xref=ChEBI:CHEBI:29035; Evidence=(PIRSR:PIRSR000106-3); Note=Divalent metal cations. Prefers magnesium or manganese. (PIRSR:PIRSR000106-3);

(Information from UniProt)

Men (UniProt:Q9U1J1) annotation
Cofactor: Mg(2+) Evidence=(PIRSR:PIRSR000106-3); Name=Mn(2+); Xref=ChEBI:CHEBI:29035; Evidence=(PIRSR:PIRSR000106-3); Note=Divalent metal cations. Prefers magnesium or manganese. (PIRSR:PIRSR000106-3);

(Information from UniProt)

men-1 (UniProt:Q9U296) annotation
Cofactor: Mg(2+) Evidence=(PIRSR:PIRSR000106-3); Name=Mn(2+); Xref=ChEBI:CHEBI:29035; Evidence=(PIRSR:PIRSR000106-3); Note=Divalent metal cations. Prefers magnesium or manganese. (PIRSR:PIRSR000106-3);

(Information from UniProt)

Alcohol dehydrogenase 4

Best gene implication in ageing for this target family came from gene A1L128 via mapping the annotation from RGD 71028 annotated in RGD 2014-03-11. Annotation GO 7568 (aging) was Inferred from Expression Pattern

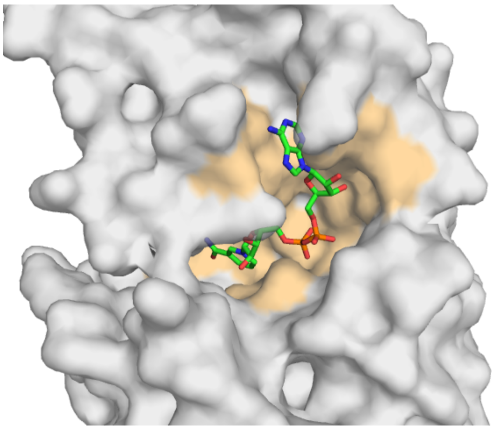

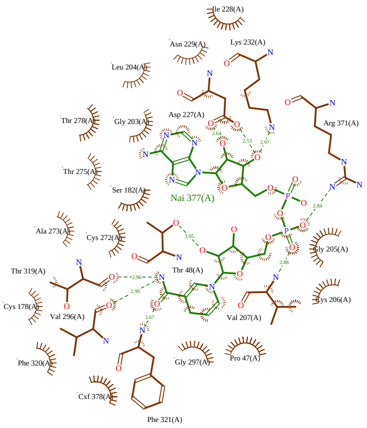

protein	amino acids contacts (binding site)																					
PDB:1e3i:chainA:Q9QYY9	P	T	C	S	G	L	G	C	V	D	I	N	K	C	A	T	T	V	G	T	F	F	R
sp:P08319:ADH4_HUMAN	H	T	C	T	G	L	G	G	V	D	I	N	K	C	A	G	T	I	G	T	F	F	R
tr:A1L128:A1L128_RAT	P	T	C	S	G	L	G	C	V	D	I	N	K	C	A	T	T	V	G	T	F	F	R
sp:Q9QYY9:ADH4_MOUSE	P	T	C	S	G	L	G	C	V	D	I	N	K	C	A	T	T	V	G	T	F	F	R
sp:P46415:ADHX_DROME	H	T	C	T	G	L	G	A	V	D	I	N	K	C	I	N	T	I	G	S	A	F	R
sp:Q17335:ADHX_CAEEL	H	T	C	T	G	L	G	A	V	D	L	I	K	C	I	N	T	I	G	T	A	F	R
tr:Q965R0:Q965R0_CAEEL	H	T	C	T	G	L	G	A	V	D	L	I	K	C	I	N	T	I	G	T	A	F	R
sp:P32771:FADH_YEAST	H	T	C	T	G	C	G	T	V	D	I	N	K	C	T	N	I	I	G	S	A	F	R

protein	whole protein ident	whole protein simil	domain-based ident	domain-based simil	contact-based ident	contact-based simil
PDB:1e3i:chainA:Q9QYY9	0.99	1.0	0.99	1.0	1.0	1.0
sp:P08319:ADH4_HUMAN	0.71	0.91	0.71	0.91	0.78	0.84
tr:A1L128:A1L128_RAT	0.93	0.98	0.93	0.98	1.0	1.0
sp:Q9QYY9:ADH4_MOUSE	1.0	1.0	1.0	1.0	1.0	1.0
sp:P46415:ADHX_DROME	0.51	0.82	0.51	0.82	0.65	0.71
sp:Q17335:ADHX_CAEEL	0.48	0.79	0.48	0.8	0.61	0.67
tr:Q965R0:Q965R0_CAEEL	0.31	0.54	0.31	0.54	0.61	0.67
sp:P32771:FADH_YEAST	0.43	0.76	0.43	0.77	0.57	0.62

Fdh (FBgn0011768) associated phenotypes
heat sensitive, memory defective

(Information from FlyBase)

Fdh (UniProt:P46415) annotation
Function: Class-III ADH is remarkably ineffective in oxidizing ethanol, but it readily catalyzes the oxidation of long-chain primary alcohols and the oxidation of S-(hydroxymethyl) glutathione.
Cofactor:Zn(2+)Note=Binds 2 Zn(2+) ions per subunit. ;

(Information from UniProt)

H24K24.3 (UniProt:Q17335) annotation
Function: Class-III ADH is remarkably ineffective in oxidizing ethanol, but it readily catalyzes the oxidation of long-chain primary alcohols and the oxidation of S-(hydroxymethyl) glutathione.
Cofactor: Zn(2+)Note=Binds 2 Zn(2+) ions per subunit. ;
Subunit: Homodimer.
Subcellular location: Cytoplasm

(Information from UniProt)

CELE$_Y50D4C.2(UniProt:Q965R0)annotation$
Cofactor: Zn(2+) Evidence=(RuleBase:RU361277);

(Information from UniProt)
